# Convergent and Divergent Paired Electrodeposition of Metal-Organic Framework Thin Films

**DOI:** 10.1038/s41598-019-50390-y

**Published:** 2019-10-04

**Authors:** Saber Alizadeh, Davood Nematollahi

**Affiliations:** Faculty of Chemistry, Bu-Ali-Sina University, Hamedan, 65174-38683 Iran

**Keywords:** Metal-organic frameworks, Sustainability

## Abstract

Employing the environmentally friendly methods for synthesis of the metal-organic frameworks (MOFs) is an urgent need and sustainable development in the synthesis of these compounds is essential. In this way, ignoring the counter electrode reaction is a potentially negative point from green chemistry standpoint which increases some issues like energy consumption and reaction time. We wish to introduce the “*paired electrodeposition*” (*PED*) technique as a new method for the simultaneous synthesis and deposition of the MOF thin films (MOFTFs). This protocol implements the uniform pattern of two MOFTF modified substrates by “*convergent* (CPED: Zn_a_/Zn_c_-MOFTFs) and *divergent* (DPED: Cu_a_/Zn_c_-MOFTFs) *paired electrodeposition*” via a one-step synthesis. With the rule of thumb, enhanced energy efficiency and atom economy, increasing electrochemical yield, time-saving along with a variety of products are advantages of this technique. Besides, the “*Electrode Modification Efficiency*” has introduced for the evaluation of functionality and modification efficiency of electrochemical heterogeneous systems, especially MOFTFs. To investigate this concept, we synthesized Zn_3_(BTC)_2_ and Cu_3_(BTC)_2_ as MOF models under constant current electrolysis in water and at room temperature. This work can make a breakthrough in the green synthesis of metal-organic frameworks.

## Introduction

MOFs that have been introduced owing to the endeavors of material scientists on the science frontier^1–3^ and followed by BASF in the industrial area^4–6^ are promising materials for the fabrication of sensor, heterogeneous catalysis, membrane, transducer, solar cell and supercapacitor in lab-on-a chip devices and scalable technologies^7–11^. Although the MOFs have been known as green materials for the sustainable goals, the eco-friendly patterning of them as a thin film with the cost-effective pricing is still challenging and open discussion^10,12–14^. Unfortunately, few studies so far have been paid attention in this issue^14^. On the other hand, fragility and insolubility of most bulk MOF crystals are deficiencies of these compounds for modification of surfaces^15–18^. To overcome these drawbacks, considerable scientific efforts have been devoted to stabilization of MOFs as thin films^16–22^. From a comprehensive perspective, two general categories can be mentioned; *ex-situ* and *in-situ* techniques^16–23^. The former methods need to transfer of synthesized MOFs onto the substrate and therefore are less acceptable than the *in-situ*, or direct growth/deposition techniques with an intergrowth and integrated thin films. Meanwhile, electrochemistry is still a niche and unique platform to address the interfacing issue of MOFs with the underlying surface^16,22,24,25^. This positive glance comes from the mild, one-step, selective and controllable strategy that would lead to the simultaneous synthesis and deposition of large-area and uniform films^16,25^. Among the electrochemistry approaches that have been dedicated toward the *in-situ*/direct modification, anodic and cathodic techniques for micro- and mesoporous MOFTFs are easy on the eyes^15,16,18,22,25–31^. In the anodic method that electrode plays the role of cation source, non-blocking of pores by salt and controlling of metal oxidation state are its beneficial features^15,16,26,29^. However, restriction on the anode material selection and single phase MOF conformation are limitations of the anodic method^15,16,26,29^. In contrast, a cathodic method with the advantages such as free-choice of electrode material, *in-situ* deprotonation of ligand, availability of cation and multiple phase MOF fabrication, has been adopted based on salt as a cation source^16,18,22,27,28,30–32^. However, the possibility of metal reduction and pore blocking by salt are the main limitations of cathodic method^16,18,22,27,28,30–32^. Alongside the distinguished advantages and disadvantages of above-mentioned methods, they have a common point which is not a critical issue at first glance, but it is a potentially negative point from green chemistry and economic standpoint. Disregarding of the counter electrode reaction is the common point which causes some remarkable issues like energy consumption, time-saving and costing to be ignored. Addressing this neglected aspect of surface electrochemistry will have significant advantages such as synchronous modification of anode and cathode, targeted electron transfer, enhanced overall energy efficiency, improved atom economy, combined electrochemical yield, time- and cost- saving, over traditional ones. This concept has been correctly employed only in the bulk solution under the title of “*paired electrosynthesis*” to the synthesis of organic and inorganic compounds^33–36^. But, to the best of our knowledge, unfortunately, this profitable aspect of heterogeneous systems, especially MOFTFs has been neglected to date. Inspired by the bulk paired electrosynthesis^33–36^ and our experience^27,37–40^, for the first example, we tried to prove the “*paired electrodeposition*” (PED) of thin layer films with a priority of the MOFTFs. To shed light on this scenario, we envisioned that the pairing of two electrochemical reactions would provide a protocol towards the fabrication of two MOFTF modified electrodes by “*convergent* (CPED) and *divergent* (DPED) *paired electrodepositions*”. In other words, by the implementation of this strategy, anodic and cathodic depositions fit together toward the two same (convergent) and two various (divergent) MOFTF modified electrodes. So, it can be an idea to take benefits of both anodic and cathodic depositions, synchronously. On the other hand, in this technique the precise matching of anodic and cathodic half-reaction is not necessary^36^. Also besides from an eco-friendly and energy consumption standpoint, it would lead to a breakthrough in the modification and functionalization of electrode and transducer surfaces by MOFTFs at the enhanced energy and time economy. This could be achieved by a one-step process at room temperature, additive base-free, green solvents and without the need for any replacement or chemical modification of the underlying surface. From a sustainable viewpoint, this narrative will involve concepts so, current efficiency, atom economy, time-saving, energy-saving, and economic issues. Besides, we have propose an “*Electrode Modification Efficiency*” (EME) parameter for evaluation of functionality, atom economy and modification efficiency for electrochemical heterogeneous systems, especially MOFTFs. In this work, we synthesized Zn_3_(BTC)_2_ ^26,41^ and Cu_3_(BTC)_2_ ^29^ MOFs through the convergent (Zn_a_/Zn_c_-MOFTFs) and divergent (Cu_a_/Zn_c_-MOFTFs) paired electrodeposition. Authors believe that the paired electrodeposition technique is a good strategy in the surface electrochemistry for the fast and synchronous modification of the conductive surfaces.

## Materials and Methods

Trimesic acid (H_3_BTC) (Merck, 95%), zinc nitrate hexahydrate (Zn(NO_3_)_2_.6H_2_O) (Sigma-Aldrich, 98%), potassium nitrate (KNO_3_) (Sigma-Aldrich, 99%), hydrochloric acid (HCl) (Merck, 37%), ethanol (C_2_H_5_OH) (Merck, 99%), were reagent-grade materials and used as received without further purification. All aqueous solutions were prepared daily with distilled water from a Millipore Milli Q water purification system at room temperature.

### Experimental procedures

#### Zn_a_-MOFTF (Anodic Electrodeposition; AED)

In typical synthesis and deposition of Zn_a_-MOFTF (based on metal), 0.127 g sodium nitrate (0.1 M) as a supporting electrolyte was dissolved in 15 mL of deionized water (solution A). Solution B was prepared by 0.525 g (2.5 mmol) of trimesic acid (H_3_BTC) dissolved in 15 mL of ethanol that added to the solution A under vigorous stirring. The precursor solution can be used for the electrodeposition process, instantly.

#### Cu_a_-MOFTF (Anodic Electrodeposition; AED)

Solution A for synthesis and deposition of Cu_a_-MOFTF (based on metal) was prepared by dissolving of 0.127 g sodium nitrate (0.1 M) as a supporting electrolyte in 15 mL of deionized water (solution A). Also, 0.525 g (2.5 mmol) of trimesic acid (H_3_BTC) dissolved in 15 mL of ethanol (solution B). Then as usual, solution B was added to the solution A under vigorous stirring and can be used for the electrodeposition process, instantly.

#### Zn_a_/Zn_c_-MOFTFs (Convergent Paired Electrodeposition; CPED)

In order to convergent paired electrosynthesis, anodic and cathodic methods have been coupled. For this goal, we used of salt and metal as two cation source. Zinc nitrate for cathodic deposition and zinc metal as a sacrificial anode to generate Zn ions for anodic deposition have employed. Solutions A and B were the same as the Zn_c_-MOFTF procedure. For this purpose, 1.33 g (4.5 mmol) of zinc nitrate as a cation source and 0.127 g sodium nitrate (0.1 M) as a supporting electrolyte were dissolved in 15 mL of deionized water (solution A, pH 2.1). Also, 0.525 g (2.5 mmol) of trimesic acid (H_3_BTC) dissolved in 15 mL of ethanol (solution B). The prepared solution aged under stirring for 2.5 h at pH 2.1 at room temperature before the electrodeposition process.

#### Cu_a_/Zn_c_-MOFTFs (Divergent Paired Electrodeposition; DPED)

Also, in order to divergent paired electrosynthesis, a homemade H-type divided two electrode cell was employed. The anodic and cathodic compartment solutions were prepared the same as Cu_a_-MOFTF and Zn_c_-MOFTF procedures, respectively for synchronous cathodic and anodic deposition. For preparation of anolyte, 0.127 g sodium nitrate (0.1 M) as a supporting electrolyte in 15 mL of deionized water was dissolved (solution A). Also, 0.525 g (2.5 mmol) of trimesic acid (H_3_BTC) dissolved in 15 mL of ethanol (solution B). Then solution B was added to the solution A under vigorous stirring. Also, for preparation of catholyte, 1.33 g (4.5 mmol) of zinc nitrate as a cation source and 0.127 g sodium nitrate (0.1 M) as a supporting electrolyte were dissolved in 15 mL of deionized water (solution A, pH 2.1). Also, 0.525 g (2.5 mmol) of trimesic acid (H_3_BTC) dissolved in 15 mL of ethanol (solution B). Then, solution B was added drop by drop to the solution A under vigorous stirring.

### Electrochemical deposition setup

Synthesis and deposition of MOFTFs based on CED, AED, and CPED techniques were performed in a homemade undivided two-electrode cell. This cell consists of a cap glass bottle containing a precursor solution, in which the working electrodes were carbon, zinc, or copper plates (20 mm × 10 mm × 3 mm) and the auxiliary electrode consisted of a stainless steel sheet. Also, we used a homemade H-type divided two-electrode cell in order to DPED technique. The all of the electrochemical synthesis and deposition experiments were done at room temperature. After preparation of the precursor solution, special working and auxiliary electrodes immersed in the precursor solution. Carbon plate and stainless steel sheet for CED (Zn_c_-MOFTF), Zinc plate and stainless steel sheet for AED (Zn_a_-MOFTF), Copper plate and stainless steel sheet for AED (Cu_a_-MOFTF), Zinc plate and Carbon plate for CPED (Zn_a_/Zn_c_-MOFTFs), Copper plate and Carbon plate for DPED (Cu_a_/Zn_c_-MOFTFs). Simultaneous synthesis and deposition of various porous metal-organic framework thin films were accomplished by applying a suitable current density for a specified period. The modified electrodes were rapidly removed from the solution and right away eluted with distilled water and ethanol. The electrodeposited film was then aged overnight at room temperature

### Instrumentation

The thin film characterizations were managed using the following instruments: The HITACHI S-4160 apparatus was employed for the recording of field-emission scanning electron microscopy (FE-SEM) images. For elemental analysis (CHN), ECS 4010 CHNSO analyzer, for inductively coupled plasma mass spectrometry, ICP MS ELAN DRC-e were employed. Powder X-ray diffraction (PXRD) patterns were recorded on an APD 2000 diffractometer in Bragg Brentano mode (2θ − θ geometry, Cu Kα1 radiation) using a linear-position-sensitive detector (SAINT-GOBain). Background corrected XRD patterns were normalized before plotting. A Pekin-Elmer GX FT-IR spectrometer was employed for Fourier transform infrared spectroscopy (FT-IR). The samples were scratched on the surface of the electrode before IR, XRD, CHN and TGA analyses. A Metrohm pH meter was utilized to measure the pH of the solutions. Synthesis and deposition thin films were performed at room temperature using a DC power supply PS-303D.

## Results and Discussion

In this research, we tried to prove the paired electrodeposition technique on the heterogeneous systems, especially for the modification of electrode surface by MOFTFs. For this purpose, we synthesized Zn_3_(BTC)_2_ and Cu_3_(BTC)_2_ MOFs for the designing of convergent and divergent paired electrodeposition concept. Qua, in CPED mode, anodic and cathodic reactions are modification of electrodes by Zn_a_/Zn_c_-MOFTF based on pertinent metal and salt as a cation source in the undivided cell, respectively. Also, in DPED mode, anodic reaction is modification of electrode by the Cu_a_-MOFTF based on corrosion of the Cu electrode surface as a cation source and cathodic reaction is the fabrication of Zn_c_-MOFTF modified electrode based on dependent salt as a cation source in the divided cell. As an additional data and obtaining comparable results, Zn_a_-, Cu_a_- and Zn_c_-MOFTF modified electrodes fabricated and characterized by the conventional cathodic an anodic electrodeposition (AED & CED) methods at our optimized conditions, individually (Figs S1–S6).

### Convergent paired electrodeposition (CPED)

**Figure 1:**
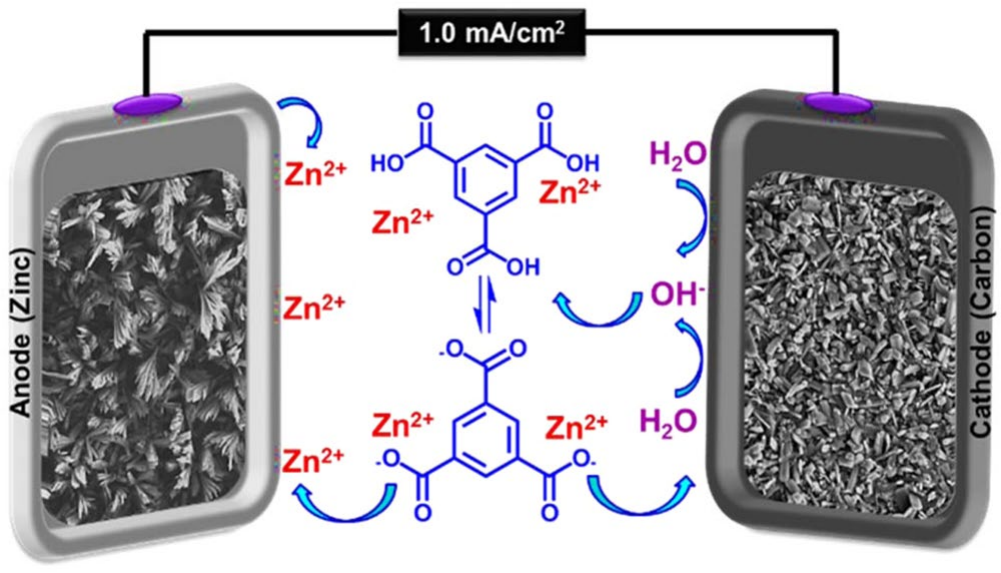
Zn_a_/Zn_c_-MOFTFs modified electrodes by the CPED.

Here, Fig. 1 presents CPED mode in order to fabrication of Zn_a_/Zn_c_-MOFTF modified electrodes through a one-step process.

**Figure 2:**
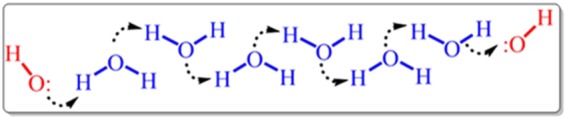
Hydroxide ion transport mechanism in aqueous solutions.

Salt and metal of zinc have employed as cation sources for the modification of the cathode and anode, respectively. The adjustment of pH is necessary for ensuring the absence of activated ligands in bulk solution and regular deprotonation at the vicinity of surface. The paired electrodeposition was started by performing constsnt current electrolysis (CCE) (1 mA cm^−2^ and 10800 s). Nucleation and growth of MOFTFs are managed on the surface without the need for any *ex-situ* base/probes at the aqueous solution with the dual task as a green solvent and hydroxide source at room temperature. The electroreduction of water generate hydroxide ions that are necessary for *in-situ* deprotonation of ligands. It should be underlined that the rate-determining step of electrosynthesis and deposition is arising local pH and consequently gradual deprotonation of ligand. The activated ligands will be coordinated to the abundantly accessible zinc cations for crystallization of Zn_c_-MOFTF on the carbon electrode surface. On the other side of the system, oxidation of zinc plate generates zinc cations which are demand for activated ligands towards the fabrication of Zn_a_-MOFTF on the zinc electrode surface. The first question that needs to be answered is the presence of deprotonated ligands in the vicinity of the anode at the steady-state conditions. The gradually increasing pH at the cathodic mode occurs only at the vicinity of cathode. Thus, it comes to mind that hydroxide ion transport from the cathode to anode should be performed via the chain hydrogen bonding at the steady state conditions (Fig. 2). The proof of the claim is the construction of Zn_a_-MOFTF on the anode and lack of formation of MOF crystals in bulk solution. It should be highlighted that in this case, generation of the cations from anode and activation of ligands at the steady state condition can be slow the crystallization rate, too.

**Figure 3:**
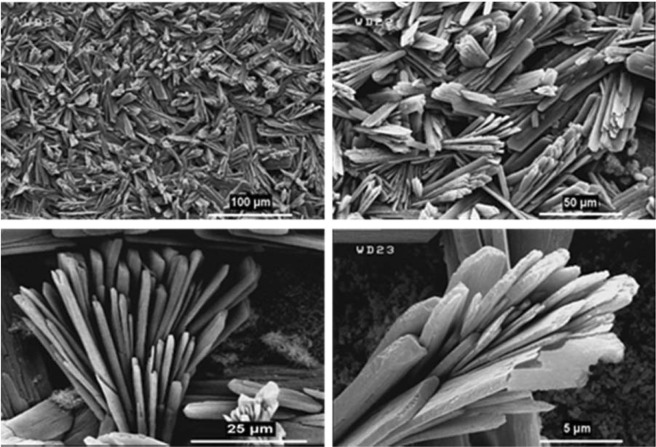
Large- and close-view FE-SEM images of Zn_a_-MOFTF modified electrode by the CPED at the I_*app*_ = 1 mA cm^−2^ and *t* = 10800 s.

**Figure 4:**
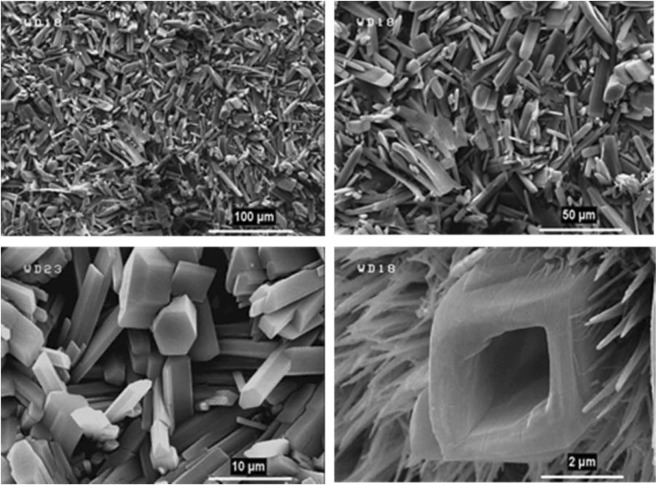
Large- and close-view FE-SEM images of Zn_c_-MOFTF modified electrode by the CPED at the I_*app*_ = 1 mA cm^−2^ and *t* = 10800 s.

**Figure 5:**
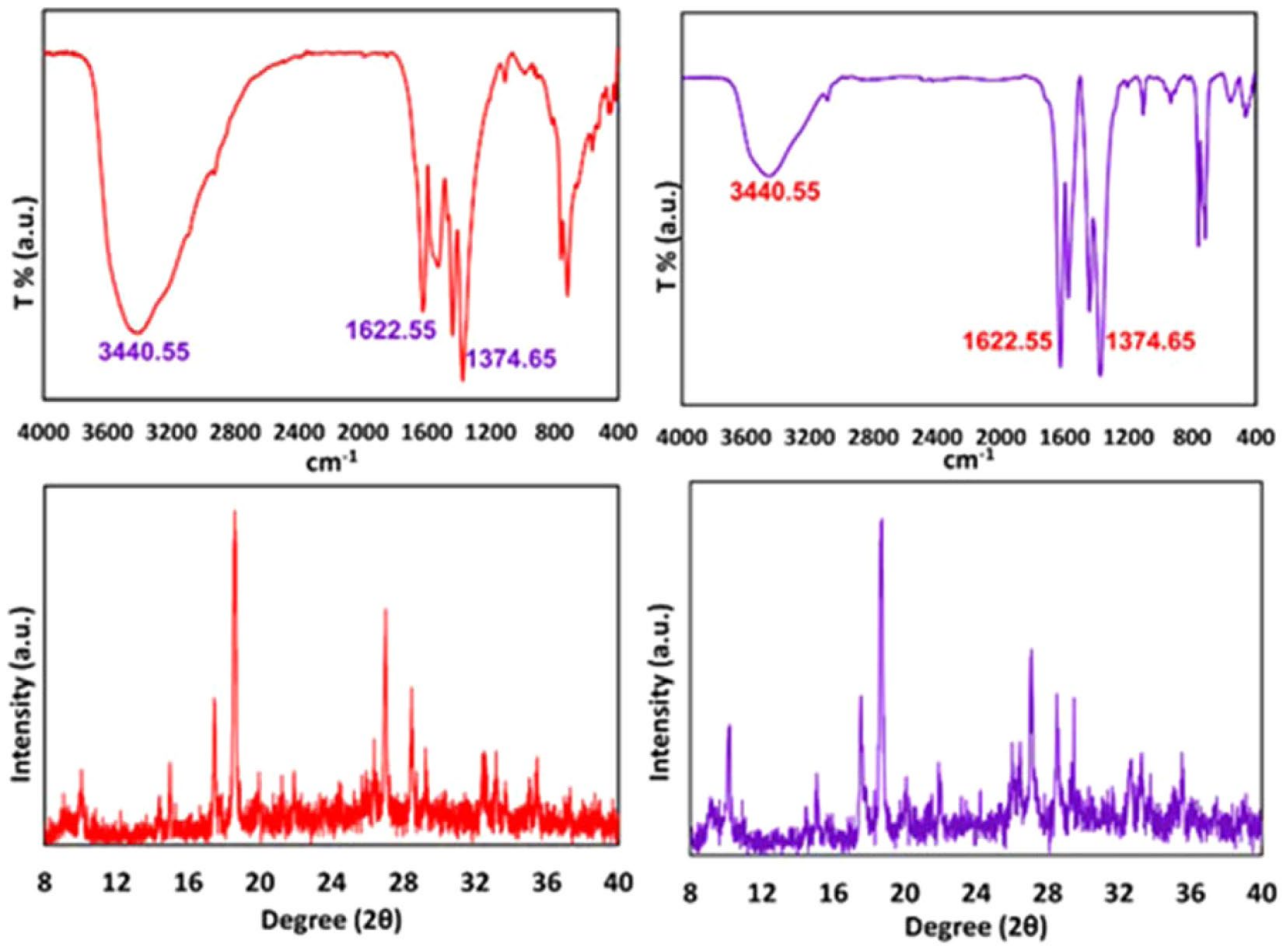
FT-IR spectra and *Ex-situ* powder X-ray diffraction patterns of scratched Zn_a_-(left) and Zn_c_-(right) MOFTFs obtained by CPED method.

These important parameters are efficient on the nucleation, growth, and arrangement of crystals on deposited film at the specified period of time. According to these reasons and what has been achieved, the anodic MOFTFs have more regular morphology than cathodic same MOFTFs. The fascinating point is the FE-SEM images of the prepared Zn- MOFTFs by CPED (Figs 3 and 4). Patterning of the continuouse hollow cylindrical cubic microcrystals and a farm of micro-shrubs grown on both sides of the carbon and zinc plates, respectively are quite similar to the conventional AED and CED methods. The analogous morphology created in CPED method is an early satisfactory certificate for the accurate performance of CPED method alongside the aforementioned benefits. The characterization of the modified electrodes was examined by FT-IR and *Ex-situ* powder X-ray diffraction (Fig. 5 and Figs S4–S9 in the SI for more discussion)^41–43^.

### Divergent paired electrodeposition (DPED)

**Figure 6:**
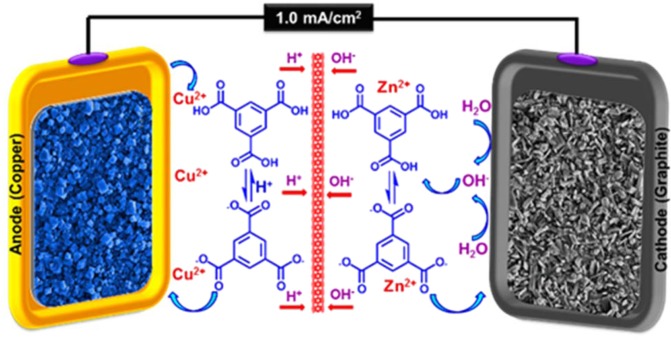
Cu_a_/Zn_c_-MOFTF modified electrodes by the DPED.

To demonstrate the high performance and generality of this protocol, we designed DPED mode in order to synchronously modification of two electrodes surface by two different MOFTFs throughout one-step electrolysis. To achieve this goal, Fig. 6 illustrates the DPED method for the synthesis of Cu_a_/Zn_c_-MOFTFs by the Zn_3_(BTC)_2_ and Cu_3_(BTC)_2_ as model MOFs, on the cathode and anode surfaces, respectively.

By starting of the CCE, hydroxide ions arising from electroreduction of water produce the activated ligands towards coordination with the zinc ions in order to crystallization of Zn_c_-MOFTF on the cathode surface. At the cathodic compartment, the adjustment of pH (2.1) is required to avoid fabrication of MOF powders in bulk solution and for installing a speed bump for slow crystallization on the substrate. From the other side, the oxidation of copper anode leads to the formation of copper ions at the vicinity of electrode surface. Unlike the catholyte, because of the absence of cation in bulk solution, the adjustment of pH for avoiding of primary deprotonation of ligands is not necessary. Nevertheless, the effective role of *in-situ* electro-generation of hydroxide ions during CCE also helps as an additional force by two roles: (a) Neutralization of the released protons from H_3_BTC/BTC^3−^ equilibrium after the reaction of BTC^3−^ with Cu^2+^ and (b) direct deprotonation of ligand. Thus, the initial solution pH is 2.8 that indicates the equilibrium of protonated form with the deprotonated form of ligands and released protons. In order to progress of the desired reaction, the free protons (with the high ionic mobility) and the hydroxide ions (with the less ionic mobility) should be pass through the sinter glace plate vice versa, by the above-explained chain hydrogen bonding for the exchanging of the above-mentioned equilibrium would lead to the activated forms. Finally, the activated ligands will be waiting for releasing cations arising from metal oxidation to crystallization of Cu_a_-MOFTF on the anodic substrate.

**Figure 7:**
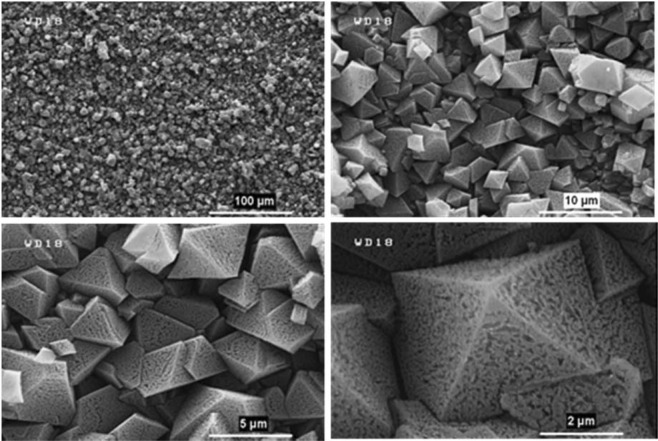
Large- and close-view FE-SEM images of Cu_a_-MOFTF modified electrode by the DPED at the I_*app*_ = 1 mA cm^−2^ and *t* = 10800 s.

**Figure 8:**
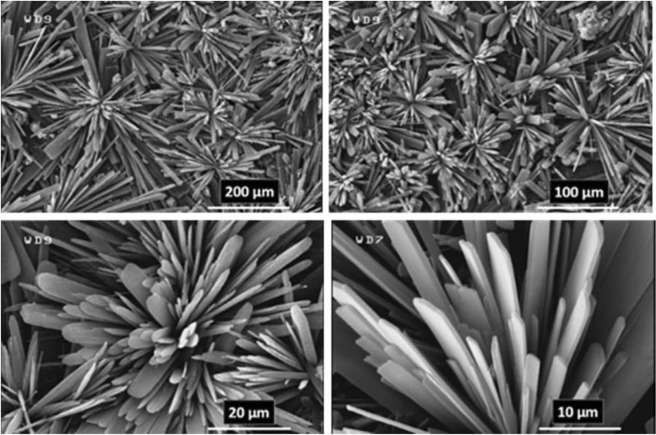
Large- and close-view FE-SEM images of Zn_c_-MOFTF modified electrode by the DPED at the I_*app*_ = 1 mA cm^−2^ *t* = 10800 s.

**Figure 9:**
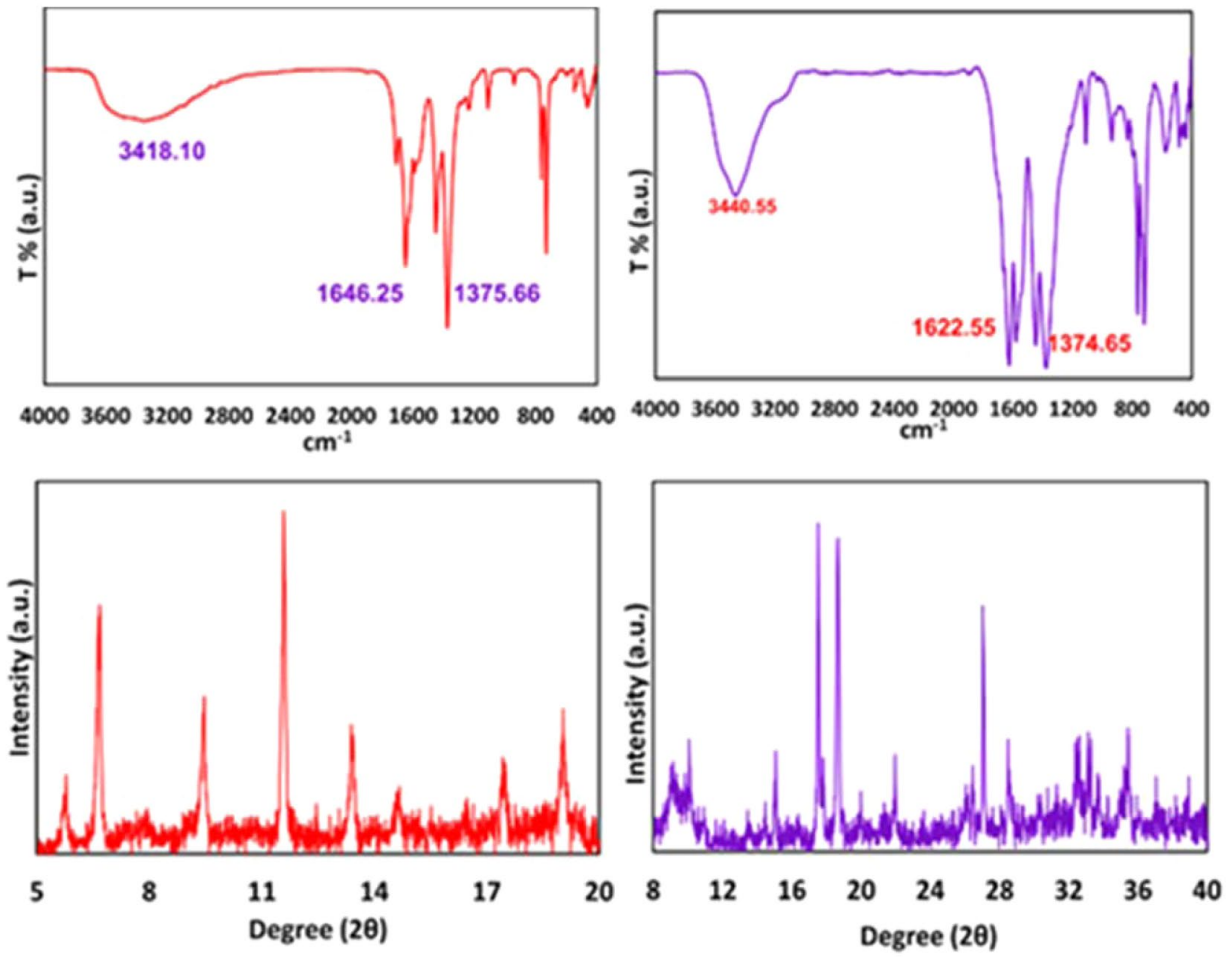
FT-IR spectra and *Ex-situ* powder X-ray diffraction patterns of scratched Cu_a_-(left) and Zn_c_-(right) MOFTFs obtained by DPED method.

As a predictable result, the FE-SEM images (Figs 7 and 8) revealed the success of DPED to the synchronous film patterning of two different types of MOFTFs on the anode and cathode surfaces via one-step CCE. The harvested grey hollow cylindrical cubic and blue sponge pyramidal microcrystals on the cathode and anode surfaces, respectively, illuminated feasibility of our vision for implementation of the PED technique. The characterization of the modified electrodes was examined by FT-IR and *Ex-situ* powder X-ray diffraction (Figs 9 and S4–S9 in the SI for more discussion)^41–43^.

It should be noted that all of the electrochemical reactions occur at the electrical double layer consists of an adsorption layer (primary or compact layer) and a secondary layer (diffusion layer). Upon the applied reductive current or potential, negatively charged cathode attracts a layer of metallic cations. Under these conditions, the secondary layer containing anions is formed on the primary layer. In this case, negatively charged ligands are present at secondary layer^44^. The reaction of metal ions at the cathode surface (compact layer) with the negatively charged ligands present in the diffusion layer results in the deposition of MOF on cathode surface.

### Electrodeposition time effect

**Figure 10:**
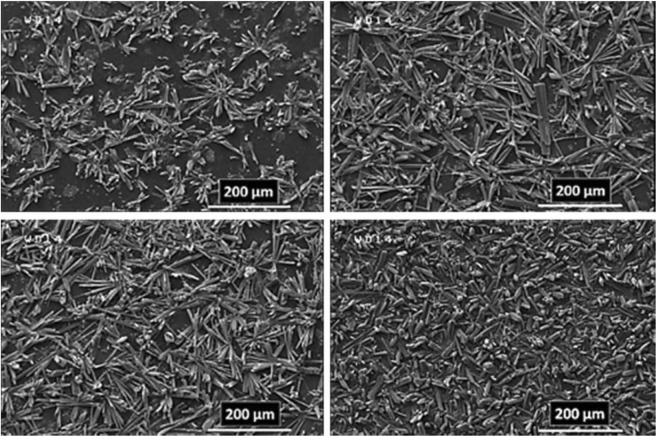
Large- and top-view FE-SEM images of Zn_c_-MOFTF modified electrode by the CPED at the I_*app*_ = 1 mA cm^−2^ and different times: t = 1800, 3600, 7200, and 10800 s.

**Figure 11:**
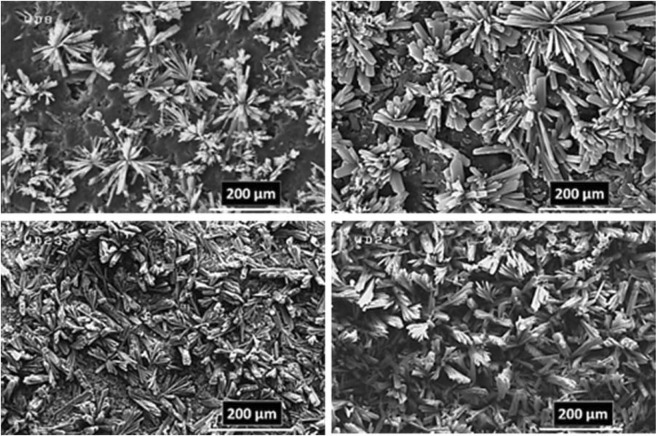
Large- and top-view FE-SEM images of Zn_a_-MOFTF modified electrode by the CPED at the I_*app*_ = 1 mA cm^−2^ and different times: *t* = 1800, 3600, 7200, and 10800 s.

**Figure 12:**
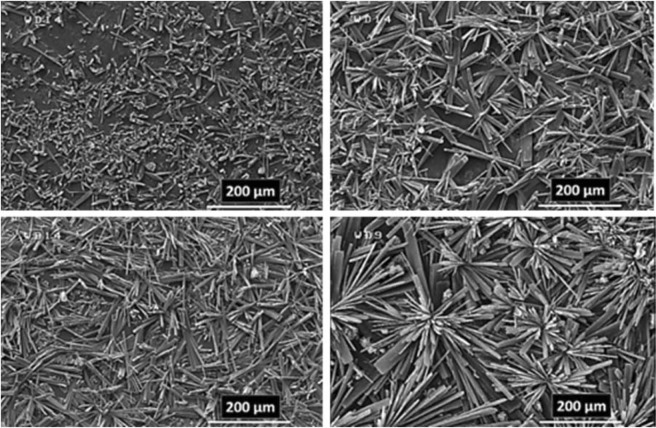
Large- and top-view FE-SEM images of Zn_c_-MOFTF modified electrode by the DPED at the I_*app*_ = 1 mA cm^−2^ and different times: *t* = 1800, 3600, 7200, and 10800 s.

**Figure 13:**
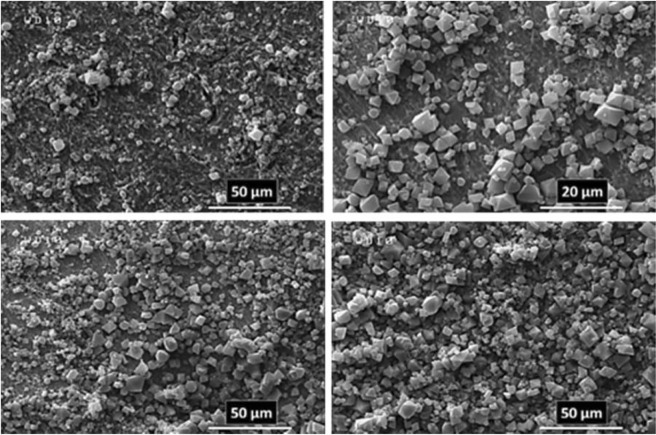
Large- and top-view FE-SEM images of Cu_a_-MOFTF modified electrode by the DPED at I_*app*_ = 1 mA cm^−2^ and different times: *t* = 1800, 3600, 7200, and 10800 s.

**Figure 14:**
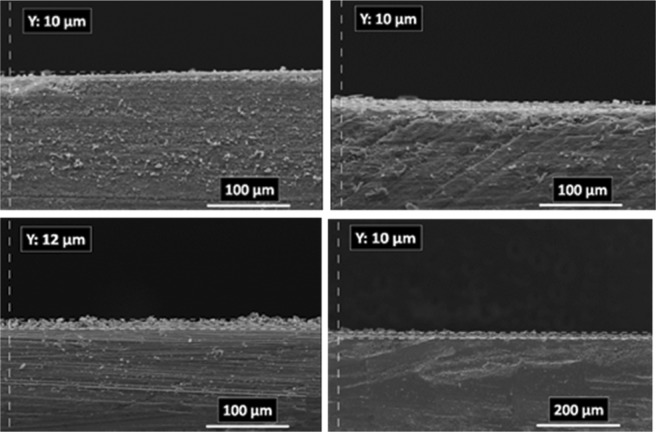
Cross section view of FE-SEM images of Zn_c_-MOFTF (up-left) and Zn_a_-MOFTF (up-right) modified electrodes by the CPED and Cu_a_-MOFTF (down-left) and Zn_c_-MOFTF (down-right) modified electrodes by the DPED at the I_*app*_ = 1 mA cm^−2^ and t = 3600 s.

**Figure 15:**
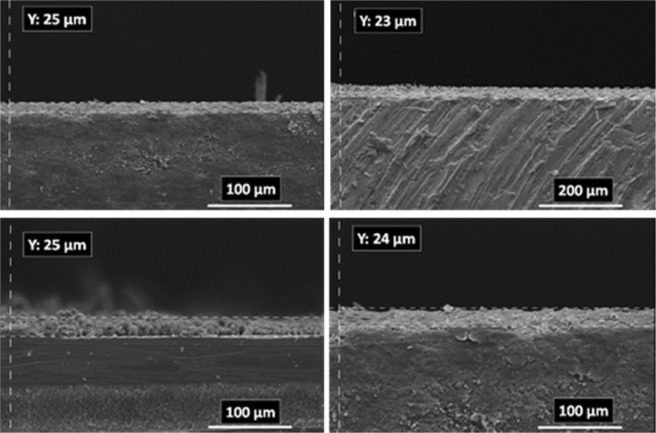
Cross section view of FE-SEM images of Zn_c_-MOFTF (up-left) and Zn_a_-MOFTF (up-right) modified electrodes by the CPED and Cu_a_-MOFTF (down-left) and Zn_c_-MOFTF (down-right) modified electrodes by the DPED at the I_*app*_ = 1 mA cm^−2^ and *t* = 10800 s.

The paired electrodeposition at different conditions provides us information on the nucleation, crystal growth and preparation of MOF thin film thickness. Figures 10–13 indicate the top view FE-SEM images of nucleation and growth of MOF crystals to full coverage of both sides of electrode surface at the different electosynthesis conditions and deposition times. Film formation could involve two processes: nucleation and growth of micro-crystals to coating the electrode surface stages. As illustrated by the images, the gaps between small crystals are sealed by highly intergrown crystals when the synthesis time is increased. In this method, since increasing electrolysis time leads to the growth and increasing size of the initial crystals, the formation of the crystals on the top of each other is less likely to occur. Also, Figs 14 and 15 (and Figs S13 and S14) show the cross-section view FE-SEM images of Zn_c_/Zn_a_ and Cu_a_/Zn_c_ MOFTFs modified electrodes. The coating thickness, the full coverage of the deposited film and crystallinity can be adjusted by the electrodeposition time. Our data show that with increasing electrodeposition time, the generation of hydroxide ion at the cathode for activation of the ligand increases. These conditions result in full and thicker coverage of the surface. In the CPED technique, the increasing thickness of the film with increasing time can be justified on the basis of more transfer of hydroxide ions from cathode to anode. Also, in the DPED technique, the increase of thickness of the films with increasing electrolysis time can be explained on the basis of more transport of electrochemically generated hydroxide ions and the charge-balancing protons through the membrane as mentioned in Fig. 6. The thickness of MOFTFs prepared at 3600 and 10800 s were determined to be 10 and 25 *μ*m, respectively.

### Current efficiency

The important point in the PED that should be considered, is current efficiency. As already highlighted, the applied current density and consequently the consumed electricity in PED is equal to the consumed electricity for the electrodeposition of MOFTFs by CED and AED methods. Figures S2, S4 and S6 illustrate that the applied electricity has been consumed only for the synthesis of MOFTF on the single electrode. Whiles, in PED the same amount electricity has been consumed for the synchronous modification of two electrodes by MOFTFs. Therefore, the current efficiency as an important part of green chemistry has been enhanced, conceptually. This parameter for CED, AED and PED methods can be calculated as shown in the Eqs 1–3.

1 $${\rm{Theoretical}}\,{\rm{mole}}\,{\rm{of}}\,{\rm{MOFTF}}=It/nF$$

2 $${\rm{Obtained}}\,{\rm{mole}}\,{\rm{of}}\,{\rm{MOFTF}}={\rm{m}}({\rm{g}}){\rm{MOFTF}}/{\rm{molecular}}\,{\rm{weight}}\,({{\rm{gmol}}}^{-1})$$

3 $${\rm{CE}} \% =({\rm{obtained}}\,{\rm{mole}}\,{\rm{of}}\,{\rm{MOFTF}}/{\rm{theoretical}}\,{\rm{mole}}\,{\rm{of}}\,{\rm{MOFTF}})\times 100$$

where *m* (g) is the weight of MOFTF (determined by weighing the bare and modified electrode before and after of the electrodeposition). Molecular weight (g mol^−1^) of MOFTF has calculated based on the molar ratio of ligand to metal that has been obtained by the ICP and CHN analysis. According to this, the CE% for the AED or CED methods is equal to 45%, while the total CE% for the CPED method is 90%. Also about the DPED mode, the calculated CE% for Cu_a_-MOFTF and Zn_c_-MOFTF in the AED and CED methods are 40% and 45%, respectively, while, the calculated CE% for DPED is 85% which is equal to the summation of anodic and cathodic calculated amounts. This important result is an expression of sustainable consumption of electricity by pre-arranged and predicted paired anodic and cathodic reactions. Also, from the time-saving standpoint, almost twice the amount of MOFTF (two modified electrodes) have been achieved in PED methods through the one-step process and the same time than AED and CED methods. Also, this methode prevents the increasing of applied potential caused by the electrogeneration of protons (electrooxidation of water) and neutralization of the electrogenerated hydroxide ions at the CED method (Please see the Fig. S12 in the SI).

### Electrode modification efficiency (EME)

Another highlighted principle of green chemistry that explains the sustainable criteria for chemical synthesis is the atom economy^34^. This concept has been well established for conventional chemical synthesis and uncomplicated compounds^35,45,46^. Unfortunately, utilizing this parameter for evaluation of efficiency and greenness of elaborate compounds and especially modification of electrodes are unclear. Recently, Freund *et al*. have been introduced an interesting parameter under the title of “*Multifunctional Efficiency*” in smart research^47^. This parameter covering four key factors: product selectivity (atom economy), specifically defined tasks (number of functional units, *FUs*), individual building units (*BUs*) and facility of production steps (*PRSs*). Finally, the authors have been formulated their hypothesis by *FR* (functionality ratio) multiplied at *PE* (process efficiency) which encompass the evaluation of functionality and easy manufacturing steps. Despite the good performance of MFE for assessment of wrapped functionalized materials, but it is not defined for the thin film modified electrodes and suffers from the lake of important electrochemical parameters. So, we have propose an “*Electrode Modification Efficiency*” (EME) parameter for evaluation of functionality and modification efficiency of electrochemical heterogeneous systems. This new equation formulated as follows (Eq. 4):

4 $$\begin{array}{ccc}EME & = & (FU\times PE)\times CE\\ EME & = & [\frac{nF{U}^{2}}{mBU\times rPRS\,}]\times [(\frac{m}{MW})/(\frac{It}{nF})]\,\end{array}$$

where *CE* is current efficieny of the employed system for the electrodeposition of MOFTFs. The value of *EME* indicates the modification efficiency of the electrode by desired system. As the first example and a better tangibility, we highlight this concept for the unpaired and paired electrochemical modification of electrode surface by the MOFTFs. In unpaired electrodeposition, in each of the Zn_c_-MOFTF, Zn_a_-MOFTF and Cu_a_-MOFTF modified electrodes, Zn_3_(BTC)_2_ and Cu_3_(BTC)_2_ have been synthesized and deposited on the cathode and anode surfaces, respectively. The above-described MOFTFs containing 2 building units (*BUs* = 2) (Zn, BTC, and Cu, BTC) which resulting in one functional unit (*FU* = 1) that have been synthesized and coated on the single electrode through the one-step reaction (*RPS* = 1), separately. The CE for the Zn_c_-MOFTF and Zn_a_-MOFTF modified electrodes was 0.45 and for the Cu_a_-MOFTF modified electrodes was 0.40, respectively. The calculated *EME* for each one of the aforementioned modified electrodes is equal to 0.225, 0.225 and 0.200 respectively, which expresses a conventional modification of the underlying substrate by the AED and CED modes. On the other hands, the CPED illustrate simultaneous synthesis and deposition of two same Zn_a_/Zn_c_-MOFTFs (*BUs* = 2, Zn and BTC) that conclude in one functional unit (*FU* = 1) on the two electrode surfaces throughout the one-step process (*RPS* = 1). The CE for the CPED system calculated 0.90 in previous section. The resulting of *EME* = 0.45 indicates the improved modification efficiency by the CPED method. At the evaluating of the DPED method, two different Cu_*a*_/Zn_*c*_-MOFTFs (*BUs* = 3, Zn, Cu, and BTC) by two functional units (*FU*s = 2) synchronously have been synthesized and deposited (*RPSs* = 1) on the anodic and cathodic electrode surfaces. The CE for the DPED system calculated 0.85 in previous section. As expected, the obtaining of *EME* = 1.13 is explanatory of the more efficiency of modification of electrodes by the DPED method.

In following, characterization of the modified electrodes were examined by FT-IR, PXRD, ICP and CHN analysis (Figs 5 and 9, also see Figs S7–S12 and Tables S1 and S2). All of the diagnostic analysis confirm accuracy and good performance of innovated paradigm for the modification of electrode surfaces with the same qualities as the conventional methods.

## Conclusions

Briefly, we succeeded to demonstrate the paired electrodeposition systems as promising methodology for attaining the sustainable achievement in the heterogeneous systems. We have proved our claim by the synchronous modification of two electrode surfaces by the *in-situ* simultaneous synthesis and deposition of two same Zn_a_/Zn_c_-MOFTFs (CPED) and two different Cu_a_/Zn_c_-MOFTFs (DPED) via a one-step synthesis by cooperation of coupled anodic and cathodic reactions. This modification of the anode and cathode surfaces was carried out while the current efficiency is twice as much as the unpaired methods. Thus, from a sustainable chemistry standpoint, enhanced energy efficiency and atom economy, increasing electrochemical yield, time-saving and variety of products are distinctive features of the proposed strategy. Furthermore, this protocol would provide a simple, fast, one-step, versatile and eco-friendly procedure that does not any need for replacement or pretreatment of the underlying electrode surface. Moreover, we propose a concept for the evaluation of functionality and modification efficiency of substrates in electrochemical heterogeneous systems. We find that introduction of PED concept in materials science and heterogeneous systems, especially MOFTFs, can be an outlook for modification and patterning of any conductive surface for electronic, photonic, microfluidic and separation applications. Optimistically, this primary platform may be employed for PED of other materials and thin films for diverse applications. Generalization of this significant conception is ongoing in our laboratory.

## Supplementary information

Supporting Information
