# Supplementary material for: Convergent and Divergent Paired Electrodeposition of Metal-Organic Framework Thin Films
Source: Sci Rep. 2019 Oct 4;9:14325. doi: 10.1038/s41598-019-50390-y (PMC6778079; doi:10.1038/s41598-019-50390-y)
Supplement: Supplementary file 1 — Supporting Information [file 41598_2019_50390_MOESM1_ESM.pdf]

Supporting Information

Convergent and Divergent Paired Electrodeposition of the Metal-Organic Framework Thin Films

Saber Alizadeh and Davood Nematollahi*

Faculty of Chemistry, Bu-Ali-Sina University, Hamedan 65174-38683, Iran

E-mail: nemat@basu.ac.ir and nematollahid@gmail.com Fax: 0098 - 813- 8257407, Tel: 0098 - 813-8282807

Contents

1	Scheme S1	Page 3
2	Description of Scheme S1	Page 4
3	Figure S1	Page 5
4	Scheme S2	Page 6
5	Description of Scheme S2	Page 7
6	Figure S2	Page 8
7	Scheme S3	Page 9
8	Description of Scheme S3	Page 10
9	Figure S3	Page 11
10	Characterization of the modified electrodes	Page 12
11	FT-IR spectra of scratched Zn_a-MOFTFs	Page 13
12	FT-IR spectra of scratched Zn_c-MOFTFs	Page 14
13	FT-IR spectra of scratched Cu_a-MOFTFs	Page 15
14	Explanation of Figures S7-S9	Page 16
15	Figure S7	Page 17
16	Figure S8	Page 18
17	Figure S9	Page 19
18	Explanation of Tables S1 and S2	Page 20
19	Table S1	Page 21
20	Table S2	Page 22
21	Figure S10	Page 23
22	Figure S11	Page 24
23	Figure S12	Page 25
24	References	Page 26

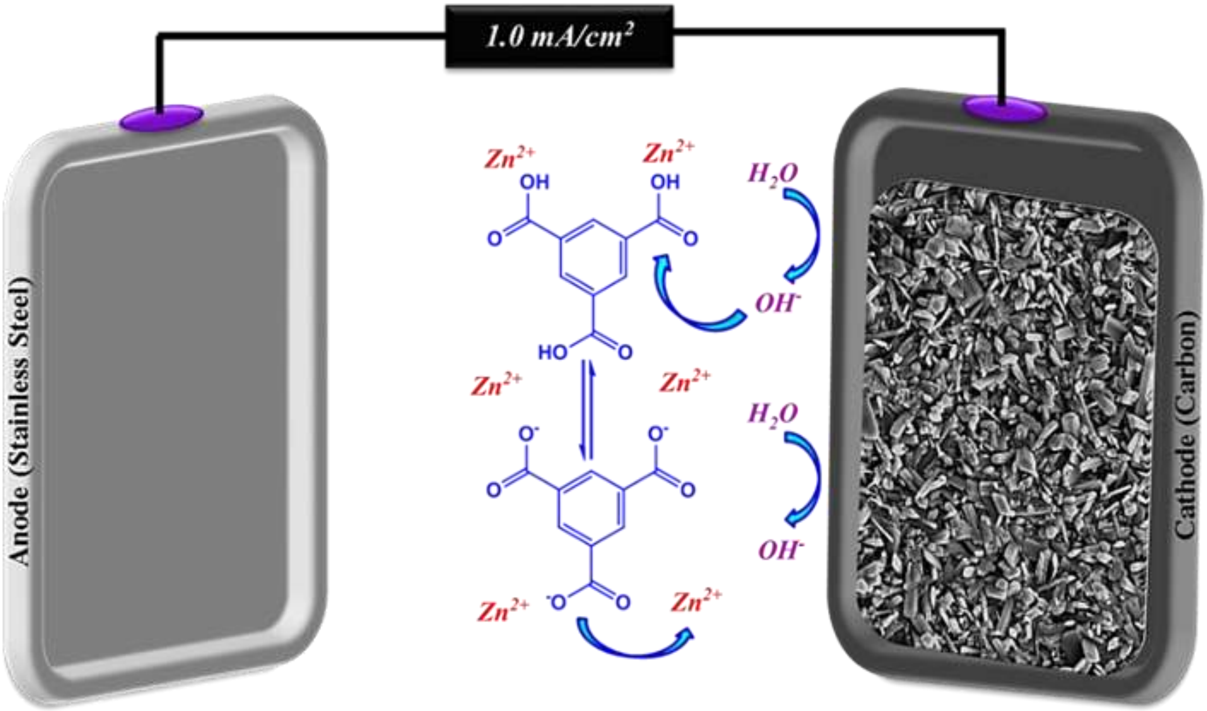

Scheme S1: Zn_c-MOFTF modified electrode by the cathodic electrodeposition (CED).

Description of Scheme S1

Scheme S1 shows the in-situ simultaneous electrosynthesis and deposition of Znc-MOFTF by the CED method. The procedure involves immersing of carbon and stainless steel as cathode and anode, respectively, in ethanol/H_2O (50:50 v/v) mixture containing $Zn(NO_3)_2$ as a cation source in the presence of H_3BTC as a ligand and $NaNO_3$ as a supporting electrolyte. The pH of the solution adjusted at 2.1 for avoiding of deprotonation of ligands and bulk crystallization of MOFs as a powder. Upon the applying constant current electrolysis (CCE) (1 mA cm^{-2} and 10800 s), the increased local pH at the cathode surface thanks to the gradual in-situ electrogeneration of hydroxide ions by electroreduction of water, make regular deprotonation of ligands. The activated ligands in the vicinity of cathode surface could be coordinated to the abundantly available cations to starting crystallinity of Zn_c-MOFTF. Eventually, nucleation and growth of Zn_c-MOFTF are managed on the cathode surface without the need for any ex-situ base/probes at the aqueous solution with the dual task as a green solvent and hydroxide source at room temperature. The gradual increase of pH in the double layer is a logical reason for intact remaining of the bulk solution. Also as a key point, the hydrogen evolution caused by electroreduction of protic solvent prevents of metal plating on the cathode electrode surface. We must keep in mind the counter electrode (anode) reaction is an indispensable reaction without any beneficial effect, here. The anodic reaction that has occurred is the oxidation of water. The electrogeneration of protons and neutralization of the electrogenerated hydroxide ions and increasing of applied potential are disadvantages of this method. It should be underlined that the rate-determining step of electrosynthesis and deposition is arising local pH and consequently gradual deprotonation of ligand. This significant parameter which controls nucleation and growth kinetics of MOF crystals can be managed by applied current and time. Figure S1 shows the FE-SEM images of the obtained Zn_c-MOFTFs on the carbon electrode surface by the CED method. Both sides of electrode exhibit full coverage of the surface with the hollow cylindrical cubic and hexagonal microcrystals with an angle approximately perpendicular to the surface.

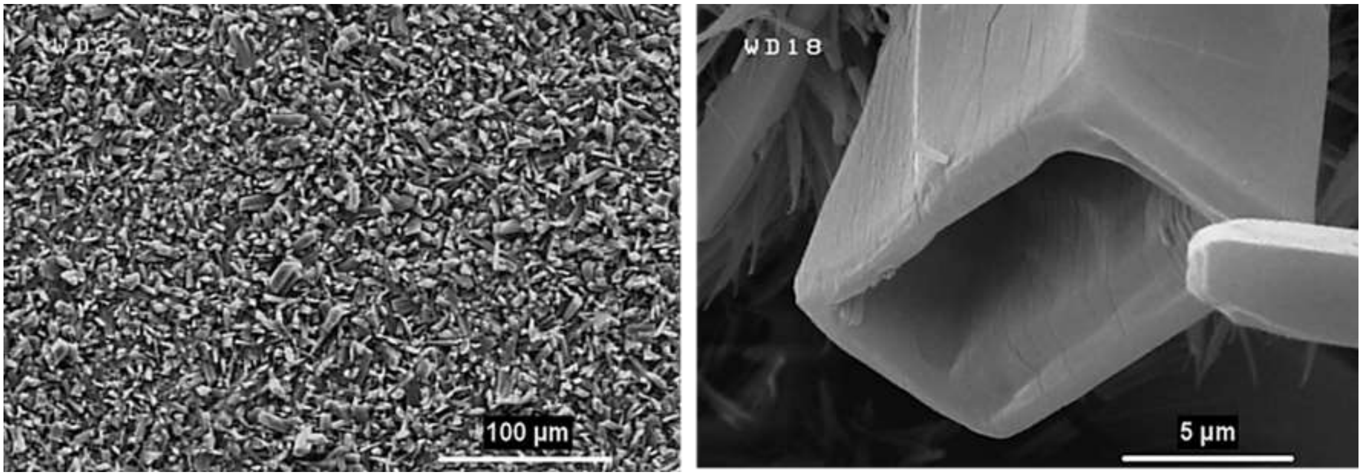

Figure S1: Large- (left) and close-view (right) FE-SEM images of Zn_c-MOFTF modified electrode surface by the CED at I_{app}= 1 mA cm^{-2} and t = 10800 s.

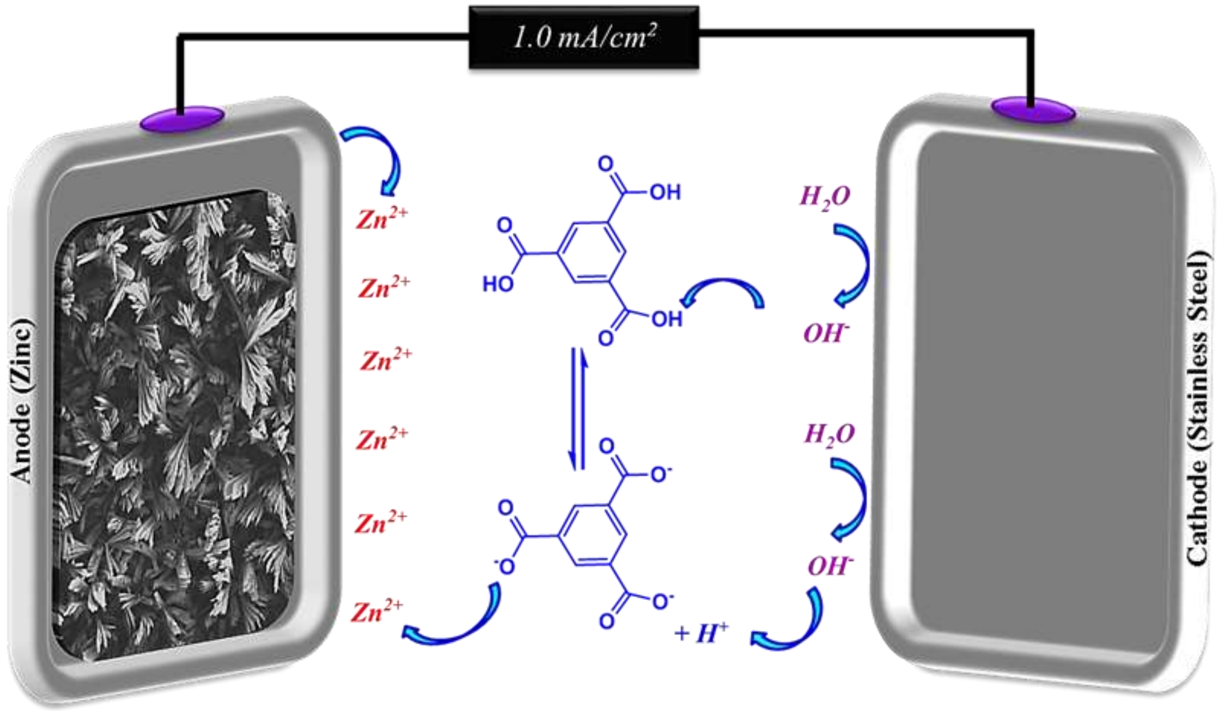

Scheme S2: Zn_a-MOFTF modified electrode by the anodic electrodeposition (AED)

Description of Scheme S2

Scheme S2 indicates the AED technique for in-situ electrosynthesis and deposition of the Zn_a-MOFTF. This mode has done by inserting of zinc metal as an anode and stainless steel as a cathode in the presence of H3BTC and $NaNO_3$ as ligand and supporting electrolyte, respectively at room temperature in an ethanol/H_2O (50/50, v/v) mixture. Zinc metal has employed as cation source and also the underlying substrate. After the establishment of suitable current density (1 mA cm^{2-} at 10800 s), the anode surface starts to oxidize in order to the preparation of zinc cations to coordinate with the available ligands. This process has some advantages and disadvantages that should be considered due to the intended application. In-situ cation electrogeneration, cation controlled release and unblocked pores of films are the positive aspects of AED method. Nevertheless, corrosion of the electrode and jagged surface, the selection constraint of the underlying substrate and deposition of single phase MOFTF are deficiencies of this procedure. It is noteworthy that an equilibrium between the H_3BTC and BTC^{3-} along with the released protons is in the electrolysis solution. Under these conditions, suggests that the electrogenerated hydroxide ions can play two roles: (a) Neutralization of the released protons from H_3BTC/BTC^{3-} equilibrium after the reaction of BTC^{3-} with Zn^{2+} and (b) direct deprotonation of ligand. It should be highlighted that in this case, generation of the cations from anode and diffusion of activated ligands from the cathode to the anode surface at the steady state condition can be slow the crystallization rate, too. These important parameters are efficient on the nucleation, growth, and arrangement of crystals on deposited film at the specified period of time. According to these reasons and what has been achieved, the anodic MOFTFs have more regular morphology than cathodic same MOFTFs. Figure S2 illustrates the FE-SEM images of Zn_a-MOFTF by AED mode that provide most regular shaped shrubs to a height of about 30 micrometers which has grown almost perpendicularly on both sides of the electrode surface.

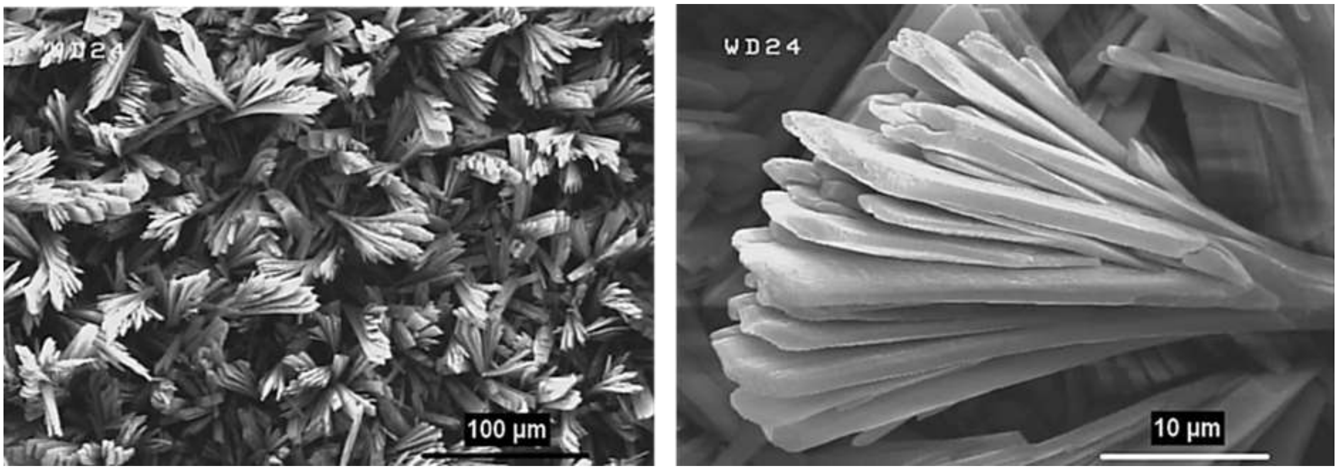

Figure S2: Large- (left) and close-view (right) FE-SEM images of Zn_a-MOFTF modified electrode surface by the AED at I_{app}= 1 mA cm^{-2} and t =10800 s.

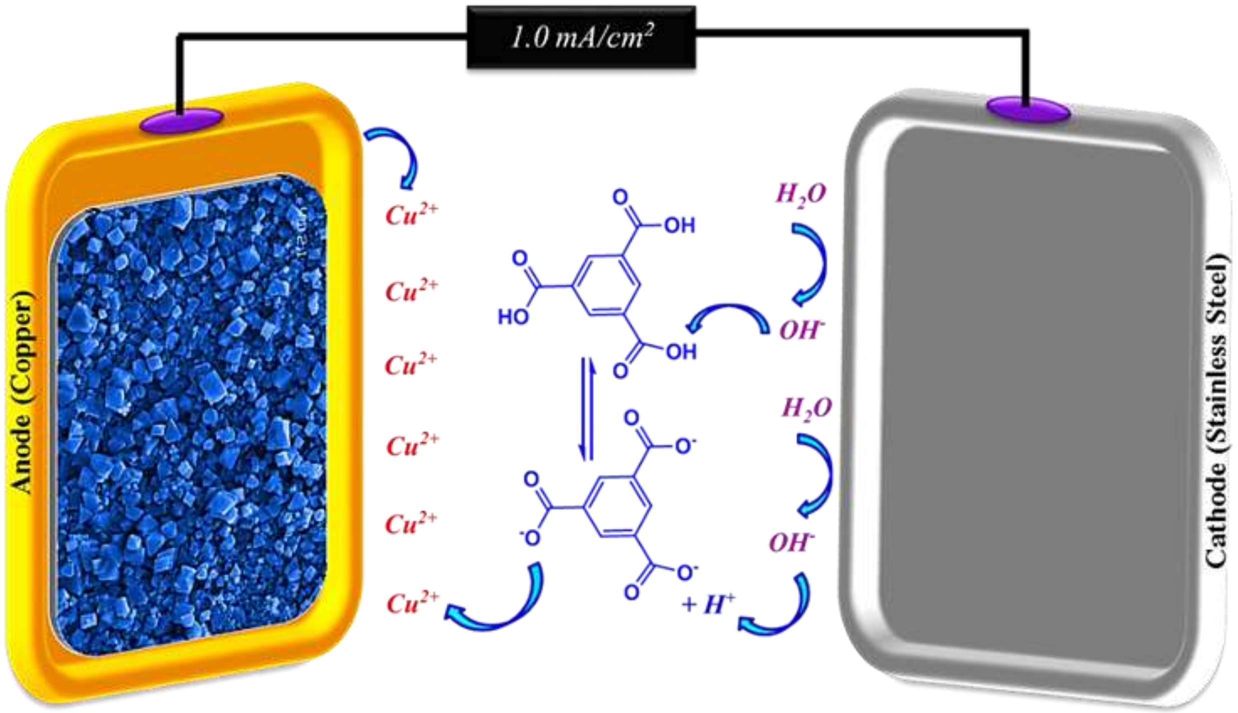

Scheme S3: Cu_a-MOFTF modified electrode by the anodic electrodeposition (AED).

Description of Scheme S3

Scheme S3 shows the pathway for the preparation of Cu_a-MOFTF by immersing of a stainless steel sheet as a cathode and a copper plate as both anode and cation source, in an undivided cell in DMF/H_2O (50/50, v/v) mixture containing H_3BTC and $NaNO_3$ as ligand and supporting electrolyte, respectively. The anodic reaction is the generation of Cu^{2+} and the cathodic reaction is the same as the AED for the Zna-MOFTF synthesis. The lack of copper ions in the bulk solution prevents bulk crystallization on the one hand, and the presence of activated ligands produced from H_3BTC/BTC^{3-} equilibrium at the surface of the anode facilities preparation of thin layer film, on the other hand. It should be noted, the neutralization of the released protons from H_3BTC/BTC^{3-} equilibrium after the reaction of BTC^{3-} with Cu^{2+} by electrogenerated hydroxide ions is necessary to progress the crystallization on the anode surface. Figure S3 indicates the full coverage of the copper plate by blue sponge pyramidal microcrystals. All of the advantages and disadvantages that have been mentioned in the preparation of Zna-MOFTF by AED method are true in this case. Here, the important point is the use of the counter electrode reaction for energy-saving and economic issues would lead to sustainable procedure.

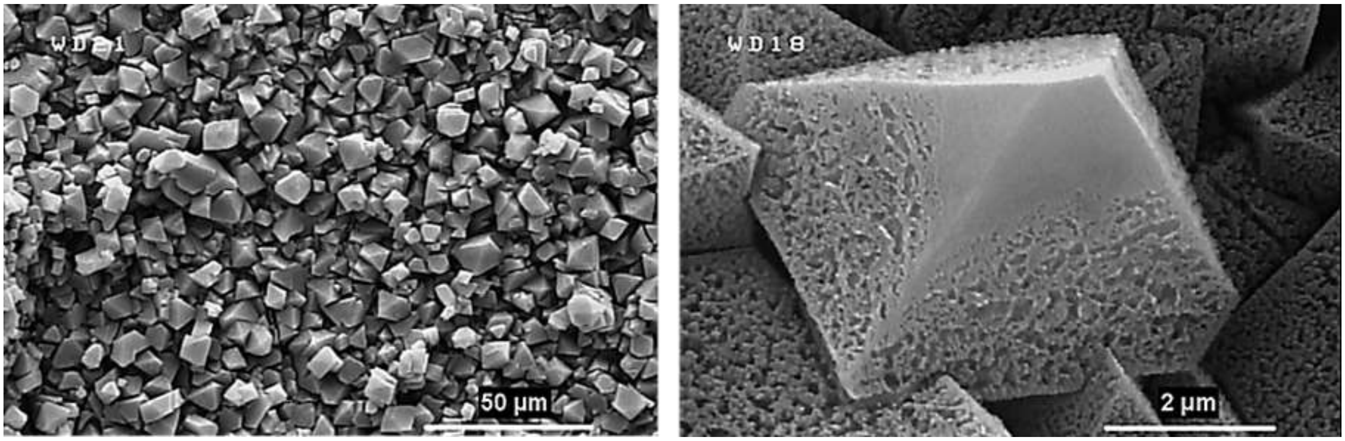

Figure S3: Large (left) and close-view (right) FE-SEM images of the by Cu_a-MOFTF modified electrode surface by the AED at I_{app}= 1 mA cm^{-2} and t =10800 s.

Characterization of the modified electrodes

In following, characterization of the modified electrodes were examined by IR, PXRD, ICP and CHN analysis (Figure S4-S9 and Table S1 and S2). Figure S4-S6 confirm the functionality and bonding groups of the scratched MOFTFs. Figure S4 and S5 present compatibilities of the scratched Zn-MOFTF by the CED (based on salt) and AED (based on metal) with the CPED and DPED techniques, respectively. Also, Figure S6 shows the consistency of the scratched Cu_a-MOFTF based on metal source by the AED and DPED techniques. The absence of widespread carboxylic acid functional group peaks (3085-2554 cm^{-1}) and the shifting of asymmetric and symmetric vibration coupled peaks of carboxylate anions (1622−1578 and 1443−1374 cm^{-1}) can be assigned to the coordination of ligands with the cations. The purity and patterns uniformity of spectra can be assigned to the unique and same reaction conditions, regardless of the applied method.[1-6]

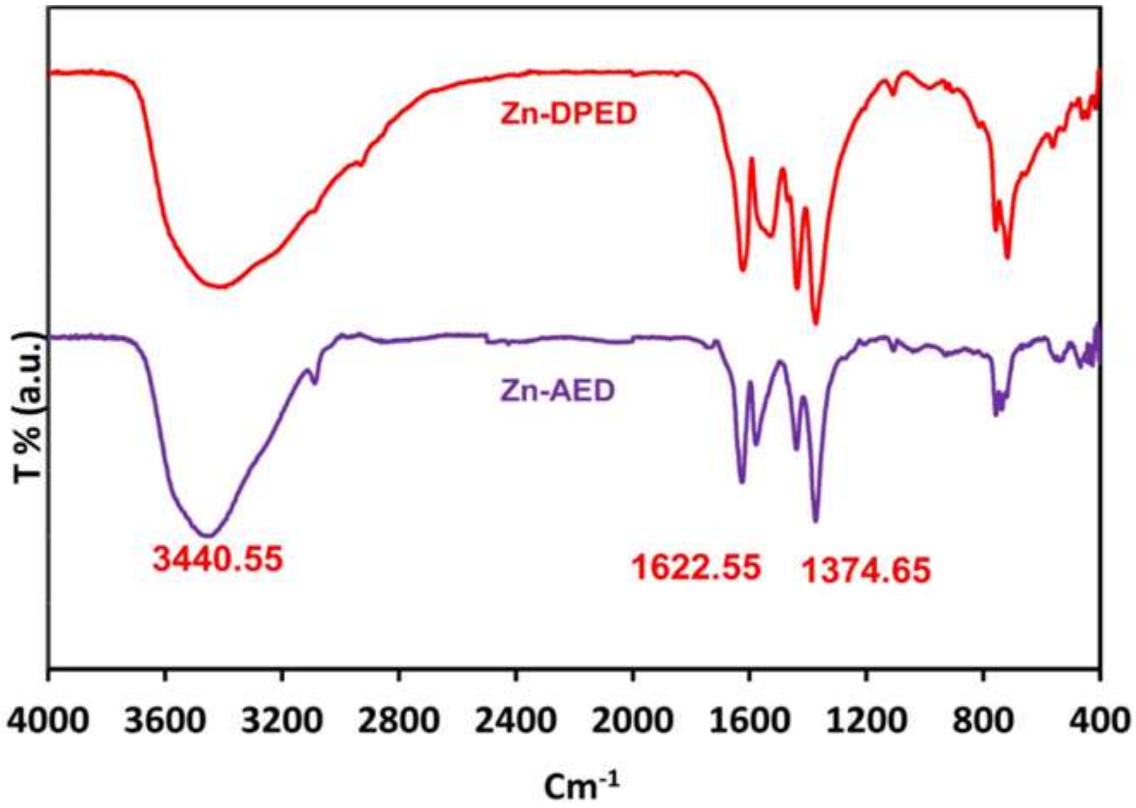

Figure S4: FT-IR spectra of scratched Zn_a-MOFTFs (based on metal) obtained by AED and DPED methods.

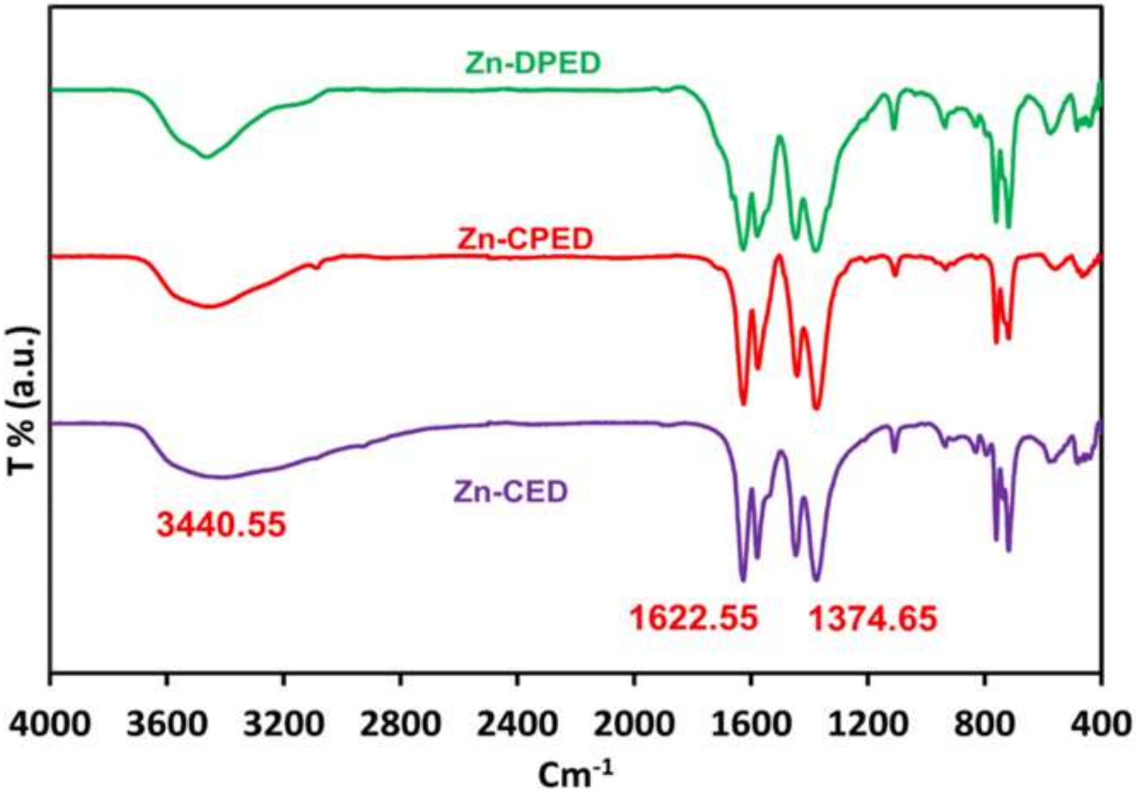

Figure S5: FT-IR spectra of scratched Zn_c-MOFTFs (based on salt) obtained by CED, CPED, and DPED methods.

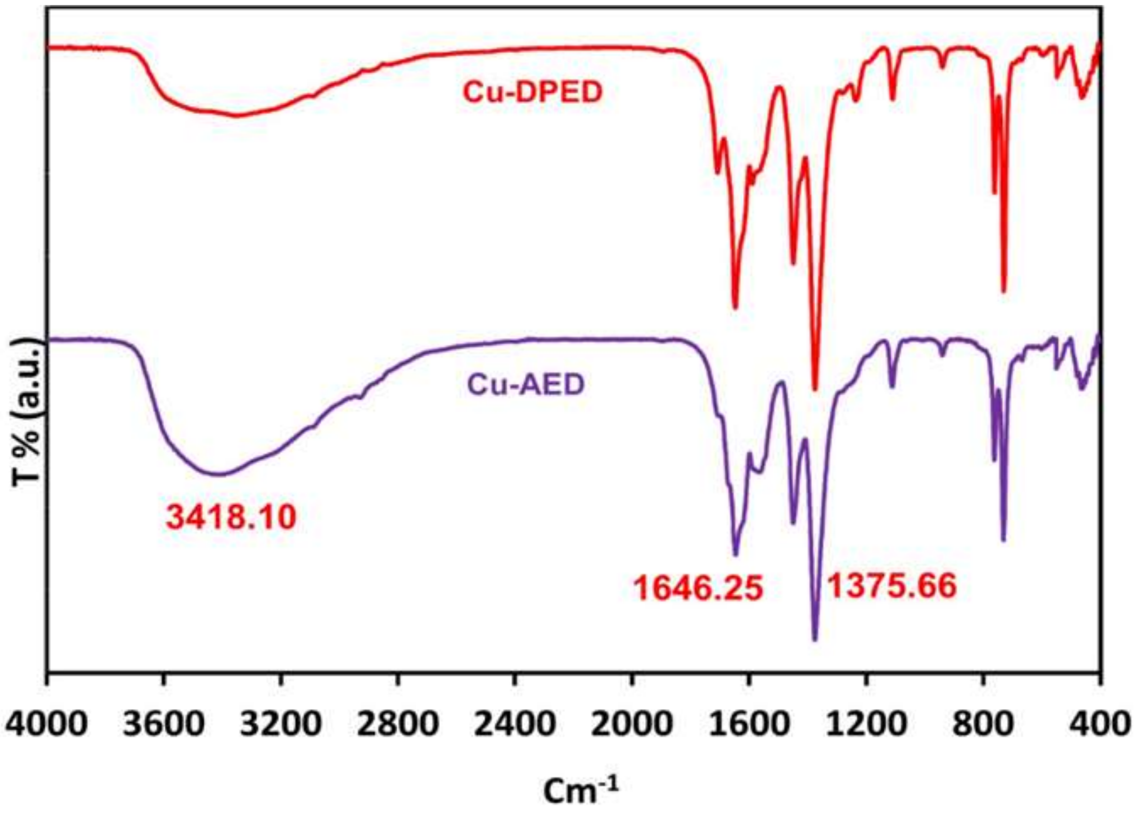

Figure S6: FT-IR spectra of scratched Cu_a-MOFTFs (based on metal) obtained by AED and DPED methods.

Explanation of Figures S7-S9

Figures S7-S9 compare the ex-situ powder X-ray diffraction patterns of the scratched various MOFTFs that have prepared by the CED and AED techniques with the prepared MOFTFs by the CPED and DPED systems. The obtained same large-angle PXRD patterns can be deducted to the well-defined crystallinity of the microporous frameworks of deposited thin films in comparison with the reported patterns,[1-6] regardless of an employed method for Zn and Cu based MOFTFs.

Based on the reported documents (Ref: 39), characteristic diffraction peaks which appeared at the 2θ 15.07° (111); 17.56° (220); 18.68° (111); 27.08° (202); 28.52° (312); 29.36° (002); 32.56° (022); 33.24° (331); 33.84° (621); 35.20° (441); 35.56° (440) confirmed that $Zn_3(BTC)_2$ has successfully synthesized with the paired electrodeposition technique. Also, Based on the reported documents (*Adv. Funct. Mater.* **2014**, *24*, 1969–1977; *Science*, **283**:5405, pg 1148-1150, 1999; J. Alloys and Compounds 540 (2012) 113–120), characteristic diffraction peaks which appeared at the 2θ 5.79° (111); 6.69° (002); 9.47° (022); 11.60° (222); 13.40° (004); 14.61° (133); 16.43° (224), 17.44° (333); 19.00° (044) confirmed that $Cu_3(BTC)_2$ has successfully synthesized with the paired electrodeposition technique. As an additional data, the absence of zinc characteristic peak declines the metal plating on the cathodic electrode surface.

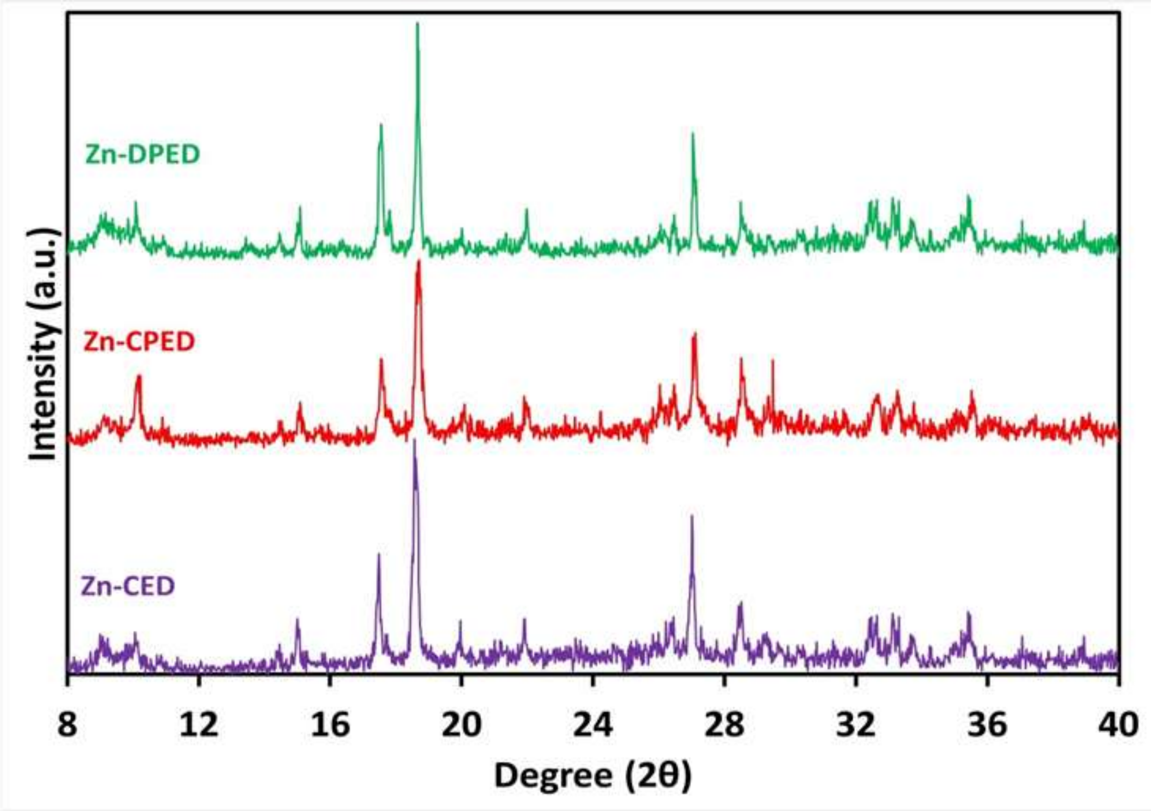

Figure S7: Ex-situ powder X-ray diffraction patterns of scratched Zn_c-MOFTFs obtained by CED, CPED, and DPED methods.

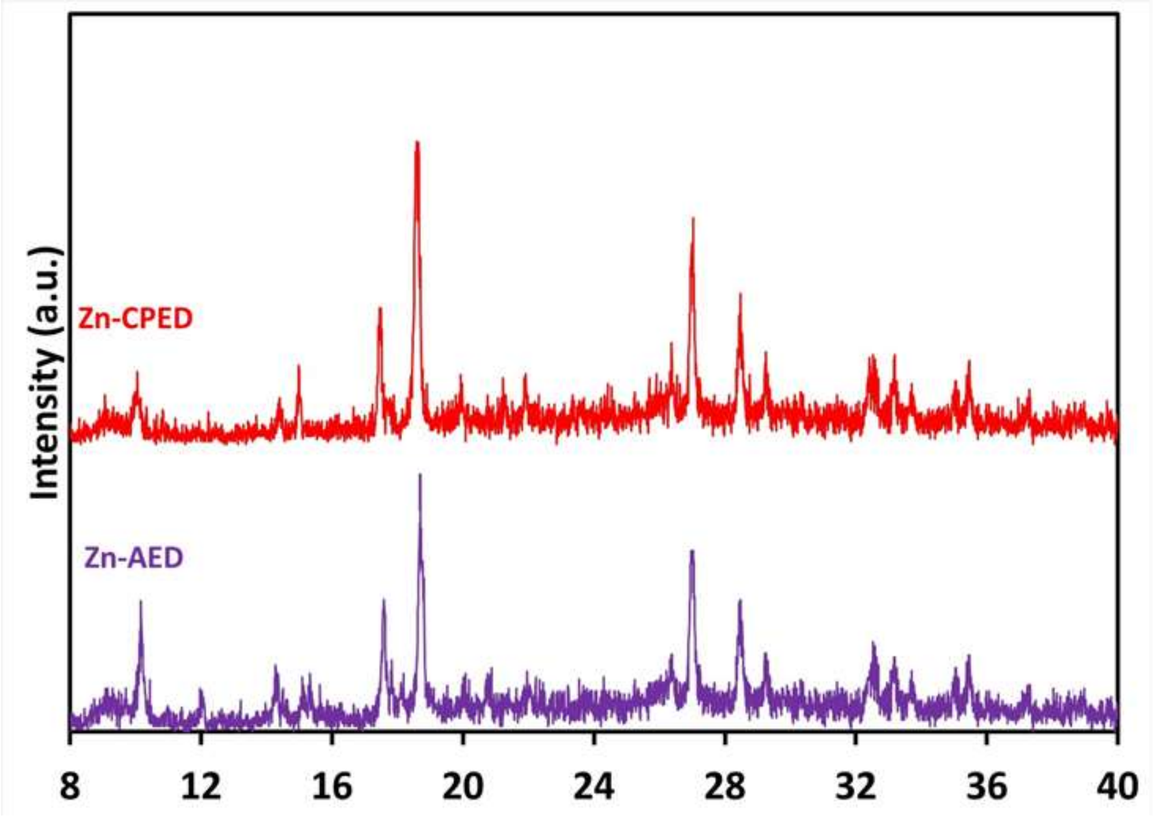

Figure S8: Ex-situ powder X-ray diffraction patterns of scratched Zn_a-MOFTFs obtained by CED and DPED methods.

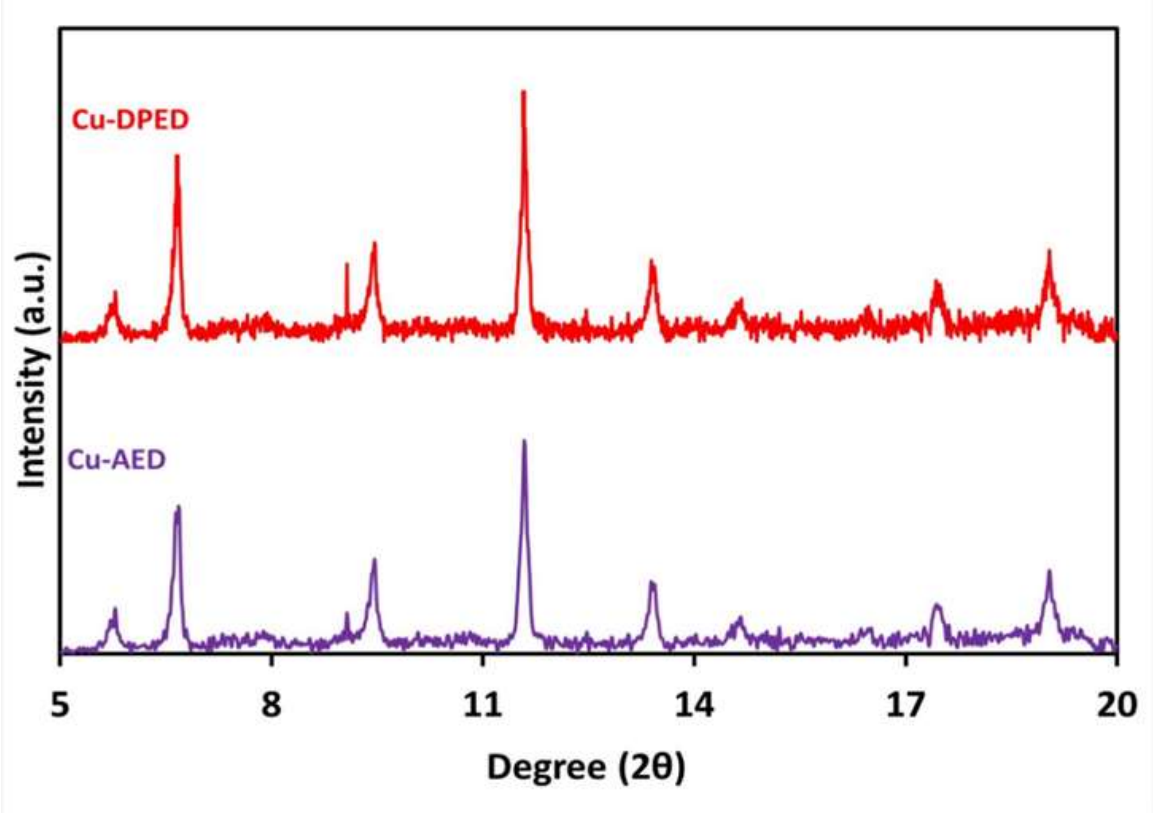

Figure S9: Ex-situ powder X-ray diffraction patterns of scratched Cu_a-MOFTFs obtained by AED and DPED methods.

Explanation of Tables S1 and S2

Tables S1 and S2 present the inductivity coupled plasma (ICP) and elemental analysis (CHN) for evaluation of ligand to cation molar ratio for the digested Zn-MOF and Cu-MOF thin films obtained by various methods. The ICP analyzed amounts of zinc for each of the five prepared electrodes are satisfactory close to the theoretical content (23.99 wt%). Also, this analysis for the value of Cu for each of the two fabricated electrodes is approximately nearby to the calculated quality (13.30 wt%). In addition due to the obtained data by CHN analysis, the calculated amounts of zinc (18.03 wt%) present the molar ratio of ligand to metal for various Zn-MOFTFs can be approximately 1:1 along with the five coordinated water molecules.[1,3] Based on these data for Cu (12.37 wt%) the molar ratio of ligand to metal for various Cu-MOFTFs estimated to be approximately 2:1 along with the two coordinated water molecules.[1,5,7]

Table S2: ICP data for various scratched MOFTFs obtained by CED, AED, CPED, and DPED methods.

**Formula**	**Element**	**Experimental**	**Theoretical**
**Zn_c-CED**	Zn_c	22.9	23.99
**Zn_a-AED**	Zn_a	24.3	23.99
**Zn_a/Zn_c-CPED**	Zn_c	23.5	23.99
**Zn_a/Zn_c-CPED**	Zn_a	25.3	23.99
**Cu_a-AED**	Cu_a	13.00	13.30
**Cu_a/Zn_c-DPED**	Cu_a	13.15	13.30
**Cu_a/Zn_c-DPED**	Zn_c	24.1	23.99

Table S3: CHN data for various scratched MOFTFs obtained by CED, AED, CPED, and DPED methods

Formula	Element	Experimental	Theoretical
**Zn_c-CED**	C/H/N/O/Zn_c	27.89/3.40/0.15/--/--	29.81/3.61/0.0/48.54/18.03
**Zn_a-AED**	C/H/N/O/Zn_a	28.35/3.64/1.09/--/--	29.81/3.61/0.0/48.54/18.03
**Zn_a/Zn_c-CPED**	C/H/N/O/Zn_c	29.11/3.52/0.22/--/--	29.81/3.61/0.0/48.54/18.03
**Zn_a/Zn_c-CPED**	C/H/N/O/Zn_a	28.35/3.50/1.07/--/--	29.81/3.61/0.0/48.54/18.03
**Cu_a-AED**	C/H/N/O/Cu_a	41.8/1.90/0.15/--/--	42.08/1.96/0.0/43.59/12.37
**Cu_a/Zn_c-DPED**	C/H/N/O/Cu_a	40.99/1.93/0.5/--/--	42.08/1.96/0.0/43.59/12.37
**Cu_a/Zn_c-DPED**	C/H/N/O/Zn_c	28.45/3.4/1.00/--/--	29.81/3.61/0.0/48.54/18.03

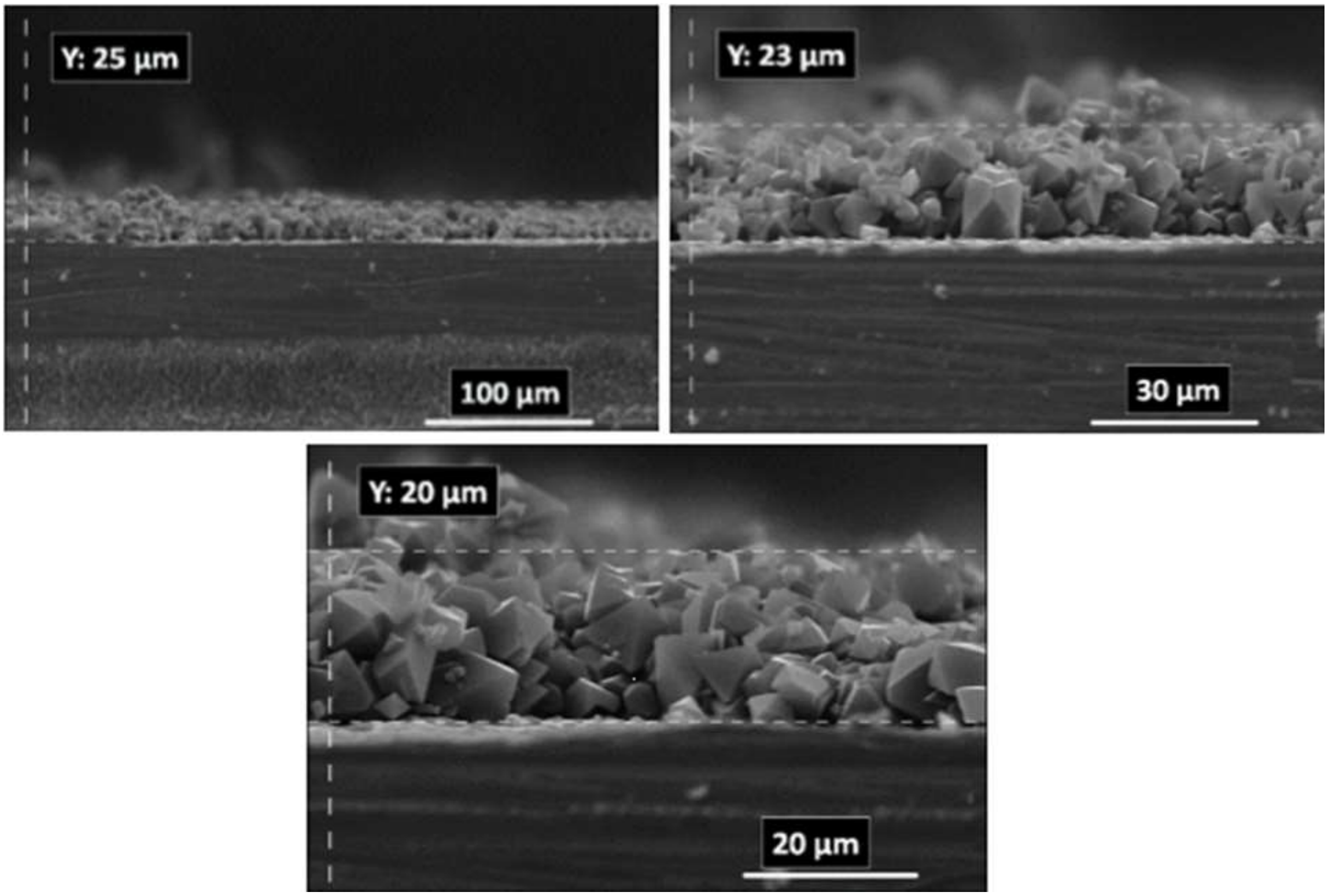

Figure S10: Cross section view of FE-SEM images of Cu_a-MOFTF modified electrode by the DPED.

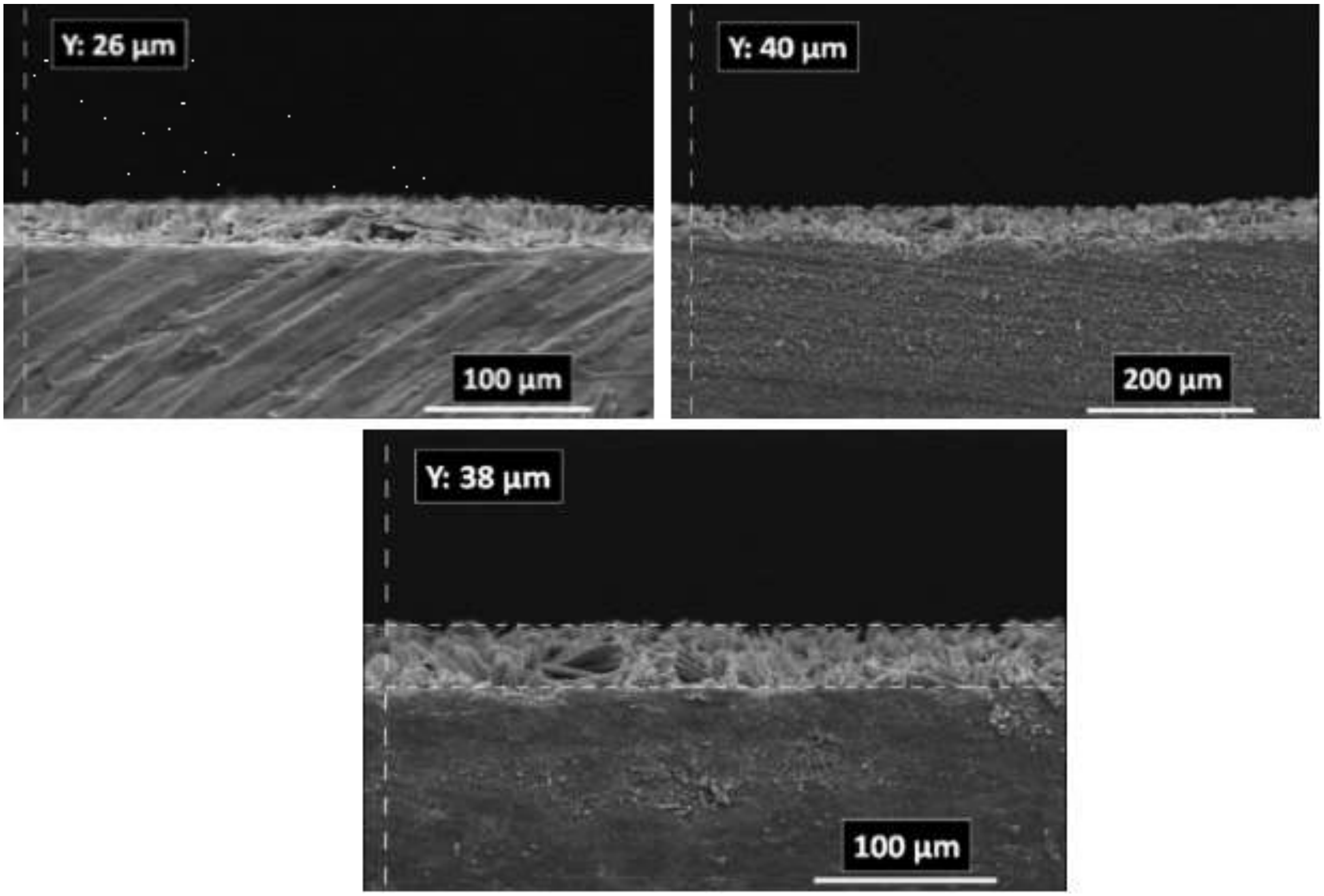

Figure S11: Cross section view of FE-SEM images of Zn_a-MOFTF (CPED) Zn_c-MOFTF (CPED) Zn_c-MOFTF (DPED) modified electrode.

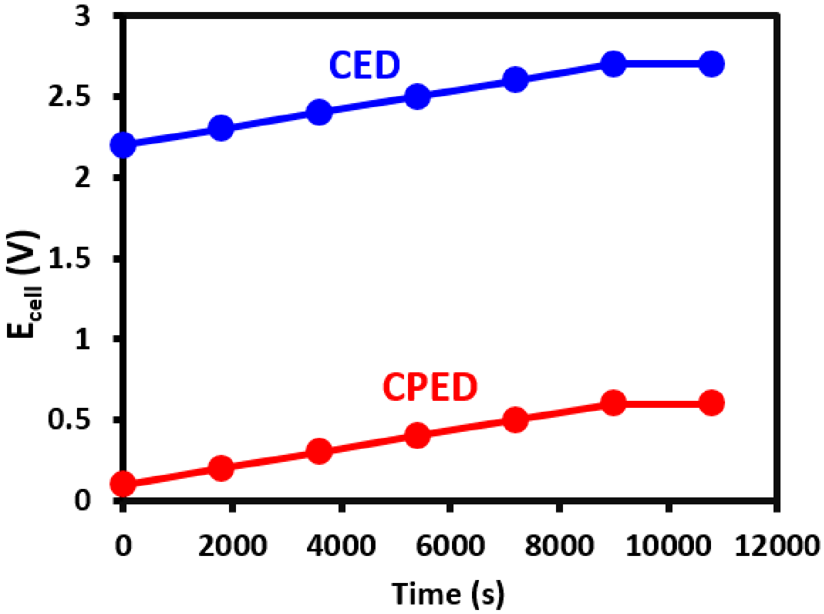

Figure S12: Evolution of cell potential vs. time during electrolysis in CED (synthesis of Zn_a-MOFTF) and CPED (synthesis of Zn_a/Zn_c-MOFTFs) techniques, at the I_{app}= 1 mA cm^{-2} and different times t = 0, 1800, 3600, 5400, 7200, 9000, and 10800 s.

The data (Figure S12) show that the pairing of cathodic electrodeposition with the anodic electrodeposition of MOF instead of the water electrooxidation prevents of the increased overpotential. Despite the low conductivity of MOFs, but the porosity of electrodeposited film makes the lower starting overpotential compared to the electrooxidation of water.

References

1. O. Yaghi, H. Li and T. Groy, *J. Am. Chem. Soc.*, 1996, **118**, 9096-9101.
2. S. Loera-Serna, M. A. Oliver-Tolentino, M. de Lourdes López-Núñez, A. Santana-Cruz, A. Guzmán-Vargas, R. Cabrera-Sierra, H. I. Beltrán and J. Flores, *J. Alloys Compd.*, 2012, **540**, 113-120.
3. S. Alizadeh and D. Nematollahi, *J. Am. Chem. Soc.*, 2017, **139**, 4753-4761.
4. Y. Wang, G. Ye, H. Chen, X. Hu, Z. Niu and S. Ma, *J. Mater. Chem. A*, 2015, **3**, 15292-15298.
5. R. Ameloot, L. Stappers, J. Fransaer, L. Alaerts, B. F. Sels and D. E. De Vos, *Chem. Mater.*, 2009, **21**, 2580-2582.
6. K. Huang, Y. Xu, L. Wang and D. Wu, *RSC Adv.*, 2015, **5**, 32795-32803.
7. R. Senthil Kumar, S. Senthil Kumar and M. Anbu Kulandainathan, *Microporous Mesoporous Mater.*, 2013, **168**, 57-64.
